# Growth kinetics of plasma-polymerized films

**DOI:** 10.1038/srep11201

**Published:** 2015-06-18

**Authors:** Sukyoung Hwang, Hosung Seo, Dong-Cheol Jeong, Long Wen, Jeon Geon Han, Changsik Song, Yunseok Kim

**Affiliations:** 1 School of Advanced Materials Science and Engineering, Sungkyunkwan University (SKKU), Suwon, Gyeonggi-do 440-746, Republic of Korea; 2 Department of Chemistry, Sungkyunkwan University (SKKU), Suwon, Gyeonggi-do 440-746, Republic of Korea

## Abstract

The growth kinetics of polymer thin films prepared by plasma-based deposition method were explored using atomic force microscopy. The growth behavior of the first layer of the polythiophene somewhat differs from that of the other layers because the first layer is directly deposited on the substrate, whereas the other layers are deposited on the polymer itself. After the deposition of the first layer, each layer is formed with a cycle of 15 s. The present work represents the growth kinetics of the plasma-polymerized films and could be helpful for further studies on growth kinetics in other material systems as well as for applications of plasma-polymerized thin films.

Recently, considerable interest has been shown in the fabrication of polymer materials for particular applications such as flexible electronic devices^1^,^2^,^3^. Accordingly, to prepare the polymer thin films, some attempts have been made using various deposition methods such as plasma sputtering, pulsed laser deposition, molecular beam epitaxy, and chemical vapor deposition^4^. A key issue for the preparation of polymer thin films is the need to understand the growth kinetics of the film. The growth kinetics of metal or oxide thin films prepared by physical or chemical deposition methods are well known, with many studies carried out^5^,^6^. In the polymer thin films, Michelmore *et al.* reported the early stage of the growth kinetics as island-like initial growth with subsequent continuous film growth^7^. However, a lack of information on the growth kinetics of polymer thin films still remains an issue. Consequently, the wide application of the preparation of polymer thin films is still restricted. In particular, even though plasma-based deposition methods such as plasma-enhanced chemical vapor deposition, among the various deposition methods, have potential for further applications of the preparation of polymer thin films, growth kinetics has thus far not been clearly understood. Plasma polymerization is a “dry” process since no solvents are used and does not require purification steps. In addition, films grown by plasma-based deposition methods can be deposited on any type of substrate, regardless of shape^4^. Thus, further application of the plasma-based deposition methods necessitates the understanding of growth kinetics in plasma-polymerized thin films. Previous reports already show that atomic force microscopy (AFM) is an excellent tool for exploring growth kinetics of film deposition as well as phase transition, etc^5^,^8^,^9^,^10^,^11^.

Here, we demonstrate the growth kinetics of plasma-polymerized thin films using AFM.

## Results and Discussion

**Figure 1:**
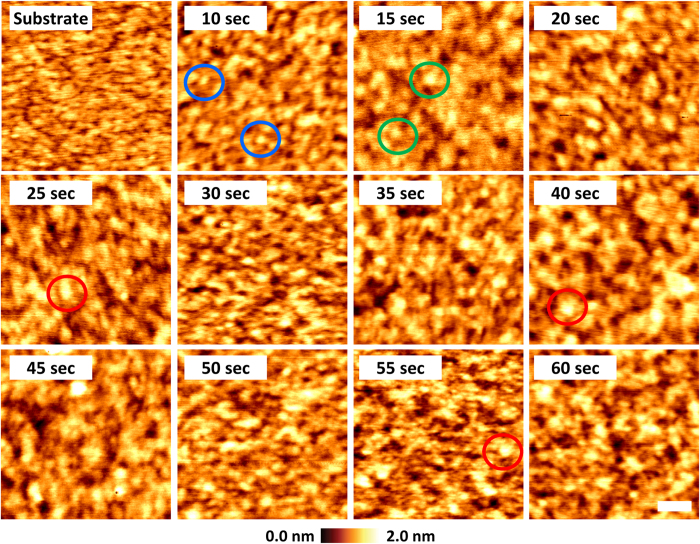
AFM topography images of substrate and plasma- polymerized films as a function of deposition time (from 10 s to 60 s with 5 s intervals). Scale bar is 50 nm.

We have examined the morphology change with increasing deposition time. Figure 1 shows AFM topography images of silicon substrate and plasma-polymerized films as a function of deposition time. As shown in Fig. 1, the topography of the substrate shows relatively low roughness, which can be inferred by the fact that most of the color in its image is within the middle range of the scale bar. After deposition of 10 s, many islands start to form and distribute on the sample surface (see blue circles in the figure of 10 s). With an increase of the deposition time to 15 s, the film surface shows fewer and slightly larger islands (see green circles in the figure of 15 s). This seems to demonstrate a smoother surface compared to the film surface in 10 s. Similar to the case in 15 s, as the deposition time further increases, the films at some of the deposition times(15, 20, 30, 35, 45, 50, and 60 s) show relatively smooth surfaces. On the other hand, the films at other deposition times show relatively rough surfaces (10, 25, 40, and 55 s). In particular, some islands are larger than the islands in these other films (see red circles). In other words, these films are relatively non-uniform.

However, growth kinetics is difficult to analyze based only on the appearance of the surface morphology. Thus, in order to systematically analyze the growth kinetics based on the obtained topography, we analyzed these data using the histogram of height distribution. The histogram of the height is extracted from the topography images (see the details in the experimental section). Since the histogram shows the distribution of the height relative to the mean value, the density of height data as well as the surface roughness can be analyzed through the shape of the histogram of height distribution.

**Figure 2:**
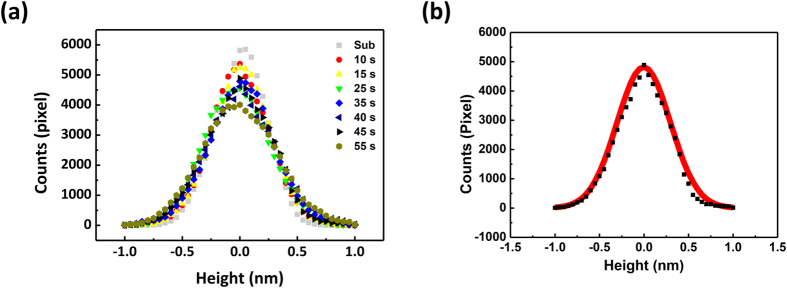
(**a**) Histograms of height distribution from selected deposition time (substrate, 10, 15, 25, 35, 40, 45, and 55 s). (**b**) Raw height data (dotted) of 45 s deposition time and corresponding fit (solid) by Gaussian function. The height data was obtained by subtracting the mean value of the raw data, which allows the peak to be aligned in the middle.

The topography images in Fig. 1 are plotted as a histogram with the height range of –1.0 nm to 1.0 nm in terms of deposition times, as shown in Fig. 2(a). Interestingly, all the data pixels are concentrated near 0 nm and are gradually distributed toward the outside. If the histogram has more than two peaks, it can be interpreted that two different growth behaviors occur, or there is an additional effect such as pieces of some particles from the target during the deposition. Since only one peak is observed, this histogram and its peak intensity can be used to estimate the changes in the surface morphology. As mentioned above, the degree of surface roughness can be analyzed by comparing the shape of the distribution in the histogram. For example, the shape of distribution in the case of the substrate is sharper than that of the other samples. This indicates the lowest degree of roughness because most pixels in the sample surface are concentrated near 0 nm. In contrast, in the case of the deposition time of 55 s, less pixels are concentrate near the center, i.e. 0 nm. Therefore, it has a higher degree of roughness. Since the obtained data is digitized, we fit the data using Gaussian function to extract the exact peak intensity for each histogram^8^. As shown in Fig. 2(b), the Gaussian function fits well with the obtained data. Thus, we further analyzed the data based on the fit using the Gaussian function.

**Figure 3:**
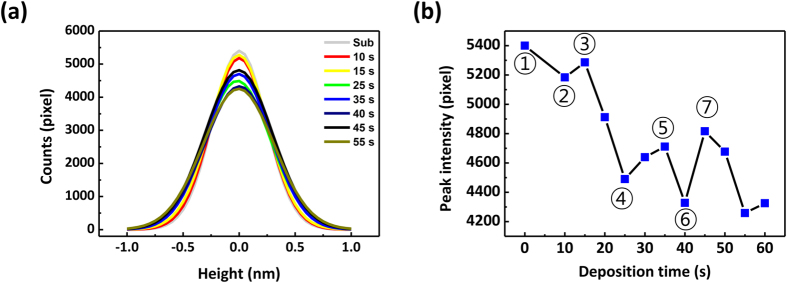
(**a**) Fit by Gaussian function for the height data in Fig. 2(a). (**b**) Peak intensity as a function of deposition time. We note that the fitting errors are negligibly small to the peak intensity. For instance, each error for 40 s and 45 s are only 0.45% and 0.42%, respectively.

Figure 3(a) shows histograms fitted by the Gaussian function at certain deposition times. The maximum value, i.e. peak intensity, in the histogram is plotted as a function of deposition time, as shown in Fig. 3(b). The peak intensity in Fig. 3(b) rapidly decreases with the increase of deposition time up to 25 s. Particularly, some points (2, 4, and 6), which show a sudden drop after small increase, periodically appear with the cycle of 15 s. This means that the peak intensity of the deposition time at that point has a periodicity and the degree of roughness suddenly increases at these points.

**Figure 4:**
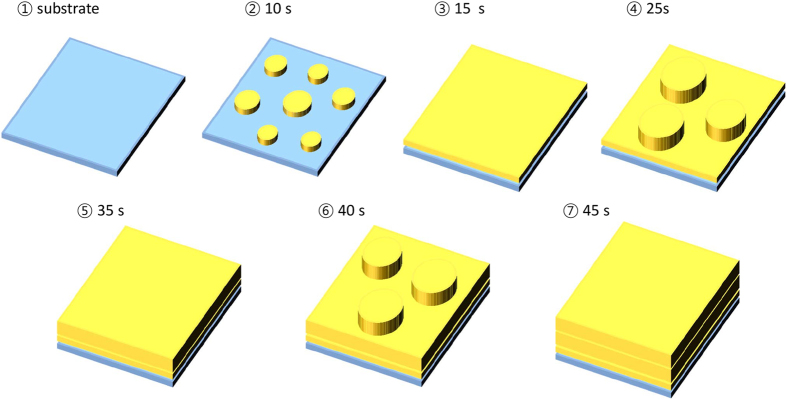
Schematic representation of growth kinetics: the number of each stage corresponds to the step in Fig. 3(b)

The growth kinetics of the plasma-polymerized films can be explained as schematically presented in Fig. 4 based on the periodicity depending on the deposition time. At the deposition time of 10 s (very early stage of deposition, 2 in Fig. 4), a number of islands consisting of aggregated polymers are formed on the substrate. A relatively low degree of roughness is then obtained at 15 s (3 in Fig. 4) compared to that of the data at deposition time of 10 s. This can be explained as the formation of the first layer. Subsequently, the sudden decrease in the degree of roughness becomes greater immediately after 15 s, and continues to increase up to 25 s (4 in Fig. 4). This corresponds to the fact that other polymer islands are created on the first layer of the polymer, which is completely formed after 15 s. Relatively lower peak intensity periodically appears at deposition times of 10, 25, and 40 (2, 4, and 6 in Fig. 4, respectively) and 55 s. However, the peak intensities at the deposition times of 10 and 25 s (2 and 4 in Fig. 4, respectively) significantly differ from each other. This might have originated from the different surface energies of the two cases; at the deposition time of 10 s (2 in Fig. 4), the polymer is deposited on the substrate; however, at the deposition time of 25 s (4 in Fig. 4), the polymer is deposited on the already deposited polymer films. If the surface energy differs, this can also affect the contact angle when the polymer is deposited on the substrate or polymer layer.

In order to obtain the surface energy of the bare silicon and thiophene thin film, the measurements of contact angle (not shown here) were performed using two different solvents, water and diiodomethane^12^. As presented in Table 1, the contact angles of water for bare silicon and thiophene thin film are 26.7 and 73.7°, respectively, and those of diiodomethane are 44.1 and 34.6°, respectively. On the basis of the contact angle information, the surface energy was calculated as 66.1 and 44.4 mJ/m^2^ for the bare silicon and thiophene thin film, respectively. Thus, since the surface energy of the substrate, i.e. the bare silicon substrate, is larger than that of the polymer, the shape of the islands on the substrate can be slightly broader and thinner than that on the polymer films. Indeed, as shown in the color circles of Fig. 1, the size of the islands seems to differ for each case. Accordingly, with the cycle of 15 s, the layer formations are completed individually. The similarity of the peak intensities of 25, 40, and 55 s and the difference between those values and that of 10 s, which is obtained when the polymer is deposited on the substrate, support this scenario.

## Conclusion

We have explored the growth kinetics of polymerized polythiophenes thin films prepared by plasma-enhanced chemical vapor deposition using AFM. To analyze the growth kinetics of plasma-polymerized thin films as a function of deposition time, we analyzed the average histogram of height distribution and peak intensities of the histogram based on the fit using the Gaussian function. The first layer shows a slightly different growth behavior to that of the other layers because the first layer is directly deposited on the substrate, whereas the other layers are deposited on the polythiophenes itself. After the deposition of the first layer, the layer formations are completed individually at every 15 s. The present work represents the growth kinetics of the plasma-polymerized films and could be helpful for further studies on growth kinetics in other material systems as well as in applications of the polymer thin films prepared using the plasma-based deposition methods.

## Experimental Methods

### Materials

Thiophene (C4H4S) was purchased from Aldrich.

### Deposition of thiophene

The plasma-polymerized films were synthesized by middle frequency (40 kHz) plasma-enhanced chemical vapor deposition using a thiophene precursor with an argon carrier gas (50 sccm). The plasma reactor chamber consists of an 8 inch top electrode and a ground electrode. The distance between the top and ground electrodes was 70 mm. Before deposition, the silicon substrates were cleaned using acetone and alcohol to remove contamination. After cleaning, the substrates were placed on the ground electrode. The thiophene was deposited for 10 to 60 s with 5 s intervals and an applied power of 4 W. During film deposition, the base pressure was approximately 1 × 10^−3^ Torr and the working pressure was 0.1 Torr. Details on the fabrication can be found elsewhere^4^.

### AFM measurements

The AFM measurements were carried out in non-contact mode using a commercial system (NX10, Park Systems). The tip used in this experiment was a silicon probe (NCHR, Nanoworld) with a radius of 8 nm and 42 N/m spring constant. The AFM topography images were first obtained with an area of 1 μm × 1 μm, consisting of 1024 × 1024 pixels. The images were then cropped to a size of 250 nm × 250 nm, consisting of 256 × 256 pixels to exclude the unexpected effect from a few particles on the surface. We averaged 6 different areas of 250 nm × 250 nm and calculated the average histograms based on the 6 histograms fitted by normal distribution of each topography image at certain deposition times.

### Surface energy

The surface energy was determined by measuring the contact angles using water and diiodomethane, and calculated using the Owens–Wendt geometric mean equation that divides the surface free energy into the dispersive γ_s_^D^ and polar γ_s_^P^ ones.


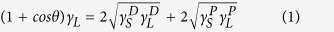


The test liquids (water and diiodomethane) slowly dropped on the surface of the test substrate material. The contact angles were measured three times for each sample and the mean value was used. The experiments were carried out at atmospheric pressure.

## Additional Information

**How to cite this article**: Hwang, S. *et al.* Growth kinetics of plasma-polymerized films. *Sci. Rep.* **5**, 11201; doi: 10.1038/srep11201 (2015).

## Figures and Tables

**Table 1:** Contact Angle Measurements and Surface Energy Calculation for a Plasma-Polymerized Polythiophene Thin Film and a Silicone Substrate.

Materials	Water contact angle (°)	Diiodomethane Contact angle (°)	Dispersive 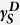	Polar 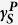	Surface energy (mJ/m^2^)
**Bare silicon wafer**	26.5	44.1	26.9	39.2	66.1
**Thiophene film with deposition time of 10** **s**	73.7	34.6	38.2	6.2	44.4
